# Enhancement of carbon monoxide concentration in atmosphere due to large scale forest fire of Uttarakhand

**DOI:** 10.7717/peerj.6507

**Published:** 2019-04-05

**Authors:** Jaya Thakur, Prajesh Thever, Biswadip Gharai, MVR Sesha Sai, VNRao Pamaraju

**Affiliations:** 1 Indian Space Research Organization, National Remote Sensing Centre, Hyderabad, Telangana, India; 2 Indian Space Research Organization, U R Rao Satellite Centre, Bengaluru, Karnataka, India

**Keywords:** Emission, Forest fire, Transport model, Trajectory, FLEXPART, Carbon Monoxide

## Abstract

The richly forested Indian state of Uttarakhand experienced widespread forest fires in April to May 2016. The current study examines dispersion of carbon monoxide (CO) from the source regions of forest fire to distant places, using the Lagrangian particle dispersion model, FLEXPART. Atmospheric Infrared Sounder (AIRS) observations revealed that CO columnar concentrations had increased by almost 28 percentage during 24 April to 02 May 2016 with respect to the previous non-burning period of April 2016 at Uttarakhand. It is also seen that there is considerable enhancement of 45 percentage in average columnar concentration of CO during the burning period, compared to that in the previous 5 years as observed by AIRS. In the present study, concentrations of CO at different pressure levels and columnar CO over Uttarakhand during the forest fire event have been simulated using FLEXPART. The area averaged profile of model derived CO has been compared with the profile from AIRS onboard Aqua. Comparison between model derived columnar CO and satellite observations shows good agreement with coefficient of correlation (*r*) approximately 0.91 over the burnt areas. Further analysis using FLEXPART reveals that the transport of pollutants is towards north-eastern and eastern regions from the locations of forest fire events. Model derived vertical distribution of CO over Tibet, which is situated at the north-east of Uttarakhand, shows significant increase of CO concentration at higher altitudes around 3 km from the mean sea level during the fire event. FLEXPART results show that the emissions from the Uttarakhand fires were transported to Tibet during the study period.

## Introduction

Forest fire is one of the major sources of air pollution, which leads to direct emission of pollutants and also formation of different constituents through secondary chemical and physical processes (Rubio et al., 2015). Forest fires lead to adverse ecological, economic and social impacts on a wide scale (Jose & Carmen, 2008; Laszlo & Rajmund, 2016; Juarez, Siebe & Fernandez, 2017). The emissions from forest fire generate huge amounts of trace gases and aerosols that have both instantaneous and long-term effects on atmosphere (Spichtinger et al., 2004). Biomass burning due to forest fires produces large emissions of carbon dioxide (CO_2_), carbon monoxide (CO), nitrogen oxides (NOx), methane (CH_4_), aerosols and other trace substances (Spichtinger et al., 2004). Pollutant emissions of CO and NOx from forest fires alter the tropospheric chemical composition, which are the precursors of tropospheric ozone (O_3_), an effective greenhouse gas itself, and may further lead to acid rain due to the production of HNO_3_ from the nitrogen cycle (Chan et al., 2003; Jaegle et al., 1998). The pollutants from such wildfires can move long distances and affect the air quality, both at the upper and lower levels of atmosphere (Damoah et al., 2004; Spichtinger et al., 2004; Wotawa & Trainer, 2000). Long distance transport of black carbon and setting over Himalayan glaciers may significantly reduce the snow albedo resulting in trapping of heat and faster melting of glaciers (Hemp, 2005; Kaspari et al., 2015). This may also alter radiation balance of the Earth atmosphere system. Hence, fires can lead to significant implications on both local and regional weather (Bytnerowicz et al., 2008). It also leads to loss of valuable timber resources, disruption of wildlife patterns and habitat and climate change.

The Indian state of Uttarakhand is richly forested (24,240 sq. km, 43.3% of the state’s geographical area (State at a Glance: Uttarakhand, FSI, 2015) as compared to 21.3% of the country’s geographical area (Executive Summary, ISFR & FSI, 2015) is forested) and comprises of the Himalayan Chir pine trees along with the broad leaved trees. Due to the shedding of pine needle shaped leaves and occurrence of resin ducts, these regions are more prone to forest fires (Manmohan & Bijalwan, 2017). The Indian states of Uttarakhand, Himachal Pradesh, Jammu and Kashmir, and the hills of Punjab, Haryana, Sikkim and North-eastern states mostly experience forest fires during the pre-monsoon season, from April to June, depending on the type of forest and climatic conditions (Manmohan & Bijalwan, 2017). Uttarakhand in general experiences forest fire events for an extended period from February to June, with a peak in the May–June period. During April 2016, number of forest fire incidents was unusually high and led to irreparable damage of valuable natural resources of the state. The worst affected districts were that of Almora, Chamoli, Nainital, Pauri Garhwal, Rudraprayag, Pithoragarh, Tehri Garhwal and Uttarkashi. Many National Sanctuaries and Reserves were reported to have been affected severely due to the forest fire event in 2016.

The number of forest fires had been considerably high in April 2016 compared to last 13 years (2003–2015) based on MODIS imagery (Jha et al., 2016). In the year 2016, Uttarakhand received scant rainfall in December and January, making it an abnormally warm winter and leading to loss of moisture from air and soil. This followed by high temperature, dry climatic conditions and dry Chir pine forest cover contributed to its severity in 2016 (Negi & Kumar, 2016). Estimation of forest fire frequency, fire potential index and fire danger model has been done by Bargali et al. (2017), Babu, Roy & Prasad (2015) and Babu, Roy & Prasad (2016) using satellite data for the Uttarakhand state. The reasons for these fires in Uttarakhand have also been examined in detail by Singh et al. (2016). Hence, examining the effect of pollutants from this forest fire on air-quality and its transport to far flung areas assumes importance. In the present study, we have examined the enhancement and transport of CO due to the forest fire event using FLEXPART and space-based observations.

Transport of CO from the Uttarakhand forest fire event duration in 2016 has been studied using FLEXPART, a long range Lagrangian particle dispersion model. Wotawa & Trainer (2000) and Joel, John & Ruben (2016) have estimated that smoke from Canadian fires led to substantial increase of CO, PM2.5 and ozone levels over USA and mid-Atlantic regions in 1995 and 2016, respectively. CO is a trace gas which is produced in large quantities during events like forest fires due to incomplete burning of biomass. It is a colorless, tasteless and odorless gas and is the most abundant air pollutant in the atmosphere exceeding the aggregate of all other pollutants (excluding CO_2_) (Louis, 1968). Its mean residence time in the lower atmosphere varies between 0.3 to 5 years and it also plays an important role as a precursor to ozone (Louis, 1968). It is known that CO plays a vital role in regulating OH in the troposphere, thereby indirectly affecting the climate, as OH is the primary removal pathway for some greenhouse gases (Streets et al., 2013). High concentration of CO leads to reduced O_2_ transport by hemoglobin in the blood and has other health effects including increased risk for people with cardiovascular problems and acute pulmonary ailments (Raub, 1999). FLEXPART has been used in the present study to examine evolution of CO, due to the forest fire event, in both space and time. It has been used to calculate the dispersion and transport of non-reactive tracers. The model has been validated extensively using large scale tracer experiments in North America and Europe (Stohl, Hittenberger & Wotawa, 1998) and performed well when compared with other models. FLEXPART has been widely used for forest fire related studies by Wotawa & Trainer (2000) and Forster et al. (2001) for Canadian forest fires and by Spichtinger et al. (2004) for Boreal and Siberian forest fires.

In the current study, validation of the model results at surface, profile and with respect to time has been carried out using space based observations from AIRS (Atmospheric Infra-Red Sounder) on-board Aqua.

## Materials and Methods

### Materials

The level 3 product of carbon monoxide from AIRS available at 1° ×1° resolution at various pressure levels up to 250 hPa and columnar values have been used in the current analysis (EARTHDATA, GES DISC & AIRS, 2018; AIRS Science Team/Joao Texeira, 2013). AIRS is a grating spectrometer on-board the second Earth Observing System (EOS) polar-orbiting platform, Aqua, and consists of atmospheric sounding group of visible, infrared, and microwave sensors. AIRS also measures abundances of other trace components in the atmosphere including ozone, carbon dioxide, methane, and sulfur dioxide, and detects suspended dust particles.

### FLEXPART trajectory model

To study the transport and dispersion of CO, we have used the FLEXPART model developed at the Norwegian Institute for Air Research in the Department of Atmospheric and Climate Research. FLEXPART is a Lagrangian Particle Dispersion Model that is used to examine long-range and mesoscale transport, diffusion, deposition, and radioactive decay of tracers released from point, line, area or volume sources in atmosphere (Stohl, Hittenberger & Wotawa, 1998; Stohl et al., 2005). Extensive validation of the model has been done (Stohl, Hittenberger & Wotawa, 1998; Cristofanelli et al., 2003; Stohl et al., 2005) and it has been widely used to evaluate the influence of various meteorological processes on pollution transport (Cooper et al., 2004; Cooper et al., 2005; Cooper et al., 2006; Hocking et al., 2007; Ding et al., 2009). For the simulation of dispersion, large numbers of particles are released from sources over the duration of emission. Backward or forward trajectories of the particles are recorded in space and time. FLEXPART can be driven by meteorological input data generated from a variety of global and regional models. A land-use inventory file (landuse.asc) has been used in the model for accounting terrain effects. Estimation of emission parameters used in the model, their temporal variation and spatial extent is discussed in detail in the ‘Results’ section.

#### NCEP FNL meteorological data

FLEXPART is an off-line model that uses meteorological fields available from ECMWF (European Center for Medium-Range Weather Forecasts), and GFS (Global Forecast System) model of the National Center for Environmental Prediction (NCEP) in GRIdded Binary (GRIB) format. In the current study, the simulations were driven by NCEP FNL (Final) global tropospheric analyses data at 1° ×1° spatial resolution and at 6- hourly temporal resolution. Parameters in FNL include temperature, u and v components of wind, vertical velocity of wind, pressure, relative humidity, planetary boundary layer height, dew point temperature, land cover, and geopotential height available at different levels like on the surface, boundary layer height, tropopause and 26 pressure levels from 1000 to 10 hPa. Data pertaining upto 250 hPa has been used in the current study for comparison with AIRS derived profile values. The data are generated from the global data assimilation system (GDAS).

#### Emission estimation

Emission rate of CO from the fire has been calculated based on experimental work done by Neto et al. (2012). The author has arrived at the emission factors for various gases like CO_2_, CO, CH_4_ and Non-Methane Hydro-Carbon (NMHC) after taking into account the average carbon content of dry biomass per unit area.

### Method

MODIS active fire locations of ∼1600 from 24 April- 04 May, 2016 have been used in the study to analyze the impact of fires. Pass-wise MODIS data are acquired and processed at the National Remote Sensing Centre, Shadnagar, Telangana using science process algorithms (SPAs) available from the Direct Readout Portal (https://directreadout.sci.gsfc.nasa.gov) for active fire products  (Giglio et al., 2003; Csiszar et al., 2014; Schroeder et al., 2014). Fire products are based on contextual fire algorithms, which rely on elevated brightness temperature values of fire pixels in middle infrared channels centered at ∼4 um against their background pixel values. Further, omission and commission errors are also considered in a series of tests to qualify a fire pixel with confidence flags (Giglio et al., 2003). The total burnt area was estimated to be 2,166 sq km (Jha et al., 2016).

Forward model run for every individual day was executed for obtaining both trajectory and concentration information at different altitudes. Outputs are obtained at every 3 h interval at different altitudes for column averaged and profile information up to 10 km height at 1 km horizontal resolution. Post-processing of the output generated was done to obtain concentration of CO at each time step by augmenting it with contribution from each individual day emission to each grid at every altitude.

## Results

**Figure 1:**
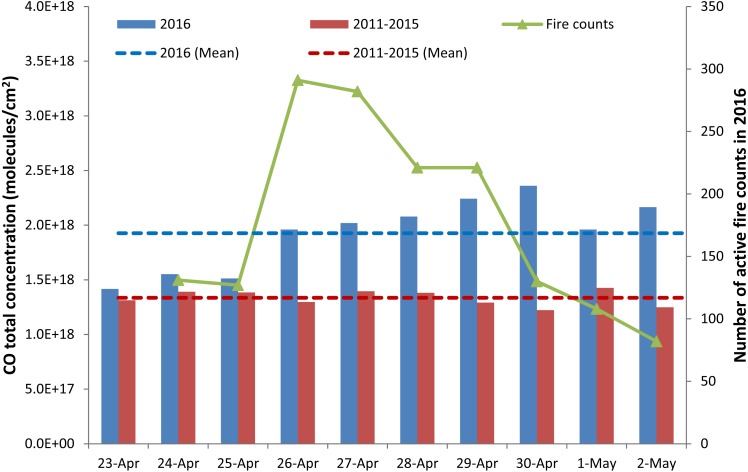
CO temporal variation with active fire counts during the event (24 April–02 May, 2016) and time series analysis of columnar CO from AIRS. Blue bars represent the columnar CO concentration for the dates in 2016. Red bars represent the average columnar CO concentration over the years 2011–2015. Blue dotted line represent the mean CO columnar concentration during the days in 2016. Red dotted line represent the mean CO columnar concentration during the days over 2011–2015. Green solid line with triangle markers represents the fire counts in 2016.

Uttarakhand witnessed more number of forest fire events in the year of 2016, compared to previous 5 years during the study period 23 April–02 May. Figure 1 shows the increase in CO concentration over Uttarakhand during the study period (compared to the corresponding daily mean of previous 5 years (2011–2015)) and the temporal distribution of fire counts during the same period in 2016. The bar chart represents the total column integrated CO concentrations over Uttarakhand from AIRS and the solid line represents the number of fire counts. It is seen that the concentrations on all days have been remarkably higher in 2016 when compared to the mean of previous years. The mean value for the 2011–2015 time period over these days is 1.3352E + 18 molecules/cm^2^, whereas it is 1.9268E + 18 molecules/cm^2^ for 2016. This shows that there was a 45% increase in the mean value of CO concentrations observed in 2016 when compared to the previous study period. Similarly, comparison with respect to the previous 9 control days non-burning period of 2016 over Uttarakhand shows an average columnar CO increase of 28% during the burning period as seen in Table 1. As seen from the figure, the maximum number of fire counts was observed on 26 and 27 April and reduced gradually up to 02 May, 2016 and the same has been simulated in FLEXPART for emission calculation. A temporal shift in concentration build-up of CO has been observed with respect to peak occurrence of number of fire counts because of longer lifetime of CO in the atmosphere.

**Figure 2:**
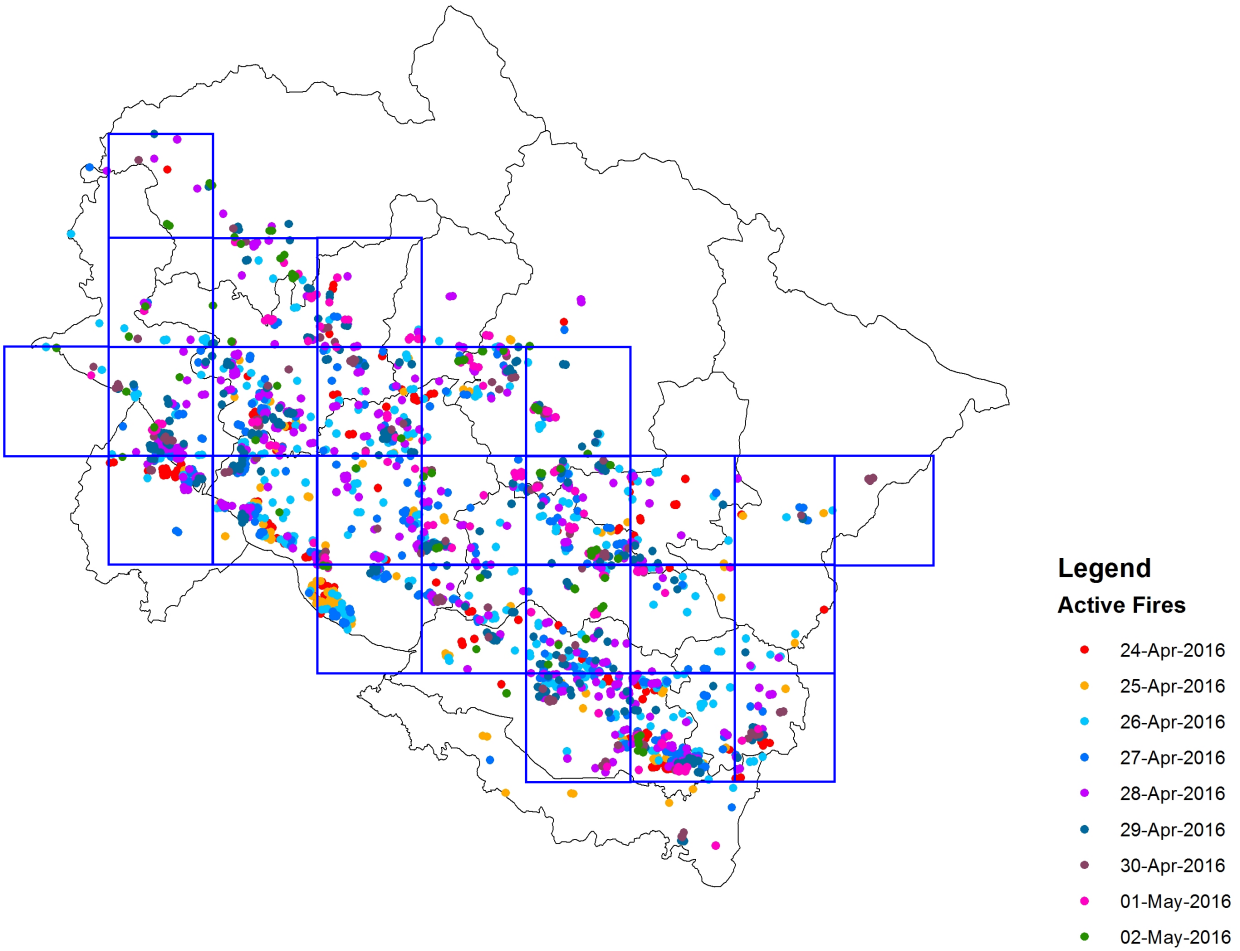
Spatial distribution of the cumulative fire counts in Uttarakhand during 24 April to 02 May, 2016 (Source: FIRMS, NASA) and emission source grids for FLEXPART analysis. Active fire locations on 24-Apr-2016 (red dots), 25-Apr-2016 (yellow dots), 26-Apr-2016 (cyan dots), 27-Apr-2016 (dark blue dots), 28-Apr-2016 (magenta dots), 29-Apr-2016 (dark green dots), 30-Apr-2016 (brown dots), 01-May-2016 (pink dots), and 02-May-2016 (light green dots).

The spatial distribution of cumulative fire counts during the study period 24 April–02 May, 2016 is shown in Fig. 2. It is seen that the fire locations are concentrated in the southern and central regions of the state. The northern side of the state of Uttarakhand is covered by the snow-covered Himalayas and hence shows lesser activity. In order to examine the spread of CO in atmosphere during the forest fire event, detailed analysis has been carried out using Lagrangian particle dispersion model, FLEXPART. Meteorological inputs for the study period have been used from NCEP FNL. The study area has been divided into 26 appropriate sized square shaped source grids of size 30 ×30 km^2^ as shown in Fig. 2, to cover the expanse of the regions burnt during the event due to the limitation of only regular shape sources in FLEXPART.

**Table 1:** AIRS CO columnar increase with respect to control days in 2016.

**Control days**		**Event days**	
Date	CO columnar concentration (mol/cm^2^)	Date	CO columnar concentration (mol/cm^2^)
14-Apr	1.51E + 18	23-Apr	1.41E + 18
15-Apr	1.49E + 18	24-Apr	1.55E + 18
16-Apr	1.54E + 18	25-Apr	1.51E + 18
17-Apr	1.57E + 18	26-Apr	1.96E + 18
18-Apr	1.17E + 18	27-Apr	2.02E + 18
19-Apr	1.42E + 18	28-Apr	2.08E + 18
20-Apr	1.92E + 18	29-Apr	2.24E + 18
21-Apr	1.49E + 18	30-Apr	2.36E + 18
22-Apr	1.47E + 18	1-May	1.96E + 18
		2-May	2.17E + 18
Mean	1.51E + 18	Mean	1.93E + 18

As mentioned in the ‘Method’ section, a total of ∼1,600 active forest fires were detected and total burnt area was estimated to be 2,166 sq km (Jha et al., 2016). The hotspot data cannot be directly used to estimate the areas burnt; hence a different methodology has to be adopted to do the same. Using the estimates of total area burnt and number of hotspots detected during the burning period, it is calculated that on an average, a hotspot covered an area of 1.35 sq km. This methodology has been used by Spichtinger et al. (2004) to calculate the burnt areas for Canadian and Siberian forest fires of 1997 and 1998. Although the hotspot areas may differ significantly from one hotspot to another, assuming that they correspond to equal areas burnt, the average hot spot area calculated has been used to determine the temporal variation of burning area within the areas identified as shown in Fig. 2. For the emissions, burnt area calculation for each single day has been done using total burnt area and number of hotspots identified on the particular day. This exercise has been carried out from 24 April to 2 May for each single day and the dispersion has been simulated up to 4 May 2016 for each individual run. Neto et al. has conducted experiments and calculated an emission rate of 24,141 kg/ha for CO, that has been used in the current study. Emissions for each day were accordingly calculated based on the burnt area (Jha et al., 2016) for the particular day and CO emission per unit area (Neto et al., 2012) to address the temporal variability in the emission using the equation

\begin{eqnarray*}\mathrm{Emission~ (kg)}=\mathrm{Burnt~ area~ (ha)}\times \mathrm{Emission~ Factor~ (kg/ ha)}. \end{eqnarray*}

To account for plume rise due to the high temperatures, it was assumed that CO emissions happen at an altitude of 300 m above the ground as suggested by Wotawa & Trainer (2000) to address the uniform spreading of gases.

**Figure 3:**
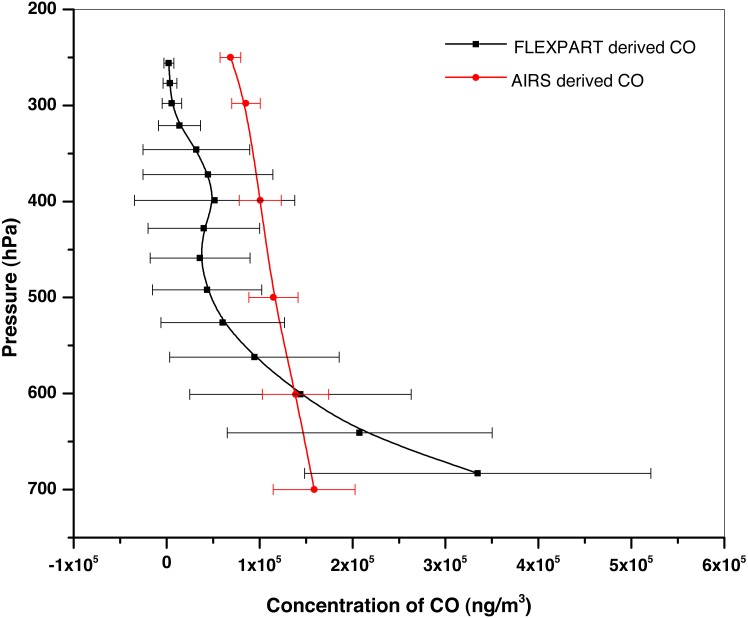
CO profile comparison of AIRS Vs. FLEXPART during the period 24 April–02 May, 2016. (A) Red line represents the CO concentration from AIRS. (B) Black line represents the CO concentration from FLEXPART.

CO concentrations at different heights at 3 hourly intervals are generated, for the period from 24 April to 2 May 2016. FLEXPART simulated CO concentrations at different altitudes have been compared with AIRS derived vertical profiles of CO up to 250 hPa at 1° ×1° resolution. Figure 3 shows the comparison of profiles during the forest fire event (24 April–02 May, 2016) over Uttarakhand. The *X*-axis represents the concentration of CO (ng/m^3^) and *Y*-axis shows the altitude pressure level (hPa). Red color line shows CO concentration from AIRS and black line represents that from FLEXPART simulations with horizontal line representing respective standard deviations. Background concentrations of CO have been removed from AIRS derived profiles, as the concentrations obtained from FLEXPART are only due to emissions from the event, whereas concentrations from AIRS are inclusive of background also. This has been done by evaluating the mean profile for the pressure levels for previous 10 non-burning days and subtracting it from the individual event day’s concentration profiles. Model run shows that model derived values of CO are higher than satellite derived at altitudes below 600 hPa. Model derived CO concentrations are mainly based on surface based inputs, hence shows much stronger response than that by satellite at lower altitudes. It is seen from the figure that the trend of vertical profile of CO concentration follows that observed by AIRS above 600 hPa. Different vertical resolutions of model and satellite derived concentration also contribute to the differences observed in slopes.

**Figure 4:**
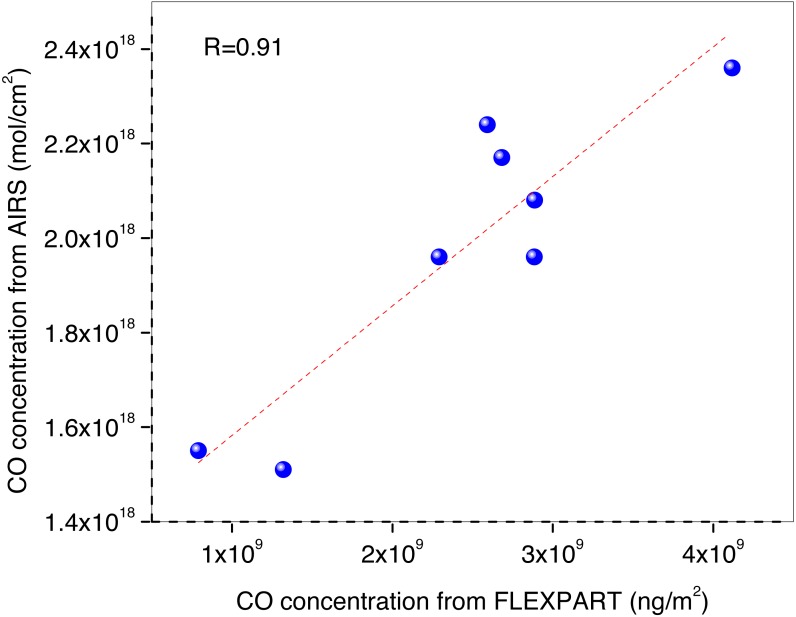
Correlation plot of Columnar CO of FLEXPART Vs. AIRS during 24 April–02 May. Blue dots represent the columnar CO concentration on each day from 24 April–02 May, 2016. Red dotted line is the best fit line between the CO concentration from AIRS and FLEXPART during 24 April–02 May, 2016.

Temporal variations of FLEXPART simulated CO concentrations have also been examined by comparing with AIRS derived concentrations for different days. Figure 4 shows scatter plot between column integrated CO from FLEXPART simulations and AIRS derived columnar CO over the study period of 24 April–02 May, 2016. Column integrated CO from FLEXPART simulations is in ng/m^2^ and that from AIRS is in molecules/cm^2^. Columnar CO from FLEXPART is correlating well with that from AIRS, with (R) ∼0.91 (with *p* < 0.02). The dots represent the value of CO concentration on each day from 24 April–02 May 2016 and the line is the best linear fit by least square method. Over the study period, the total columnar integrated CO from FLEXPART derived columnar values up to 250 hPa with AIRS columnar values, averaged over the Uttarakhand area correlate well, hence validating the results obtained by FLEXPART.

**Figure 5:**
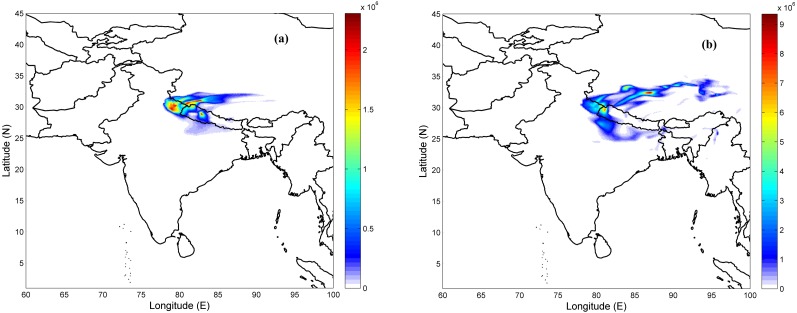
FLEXPART generated CO concentration at 500 m altitude above ground level on (A) 24 April, 2016 2100 UTC (B) 28 April, 2016 1500 UTC.

**Figure 6:**
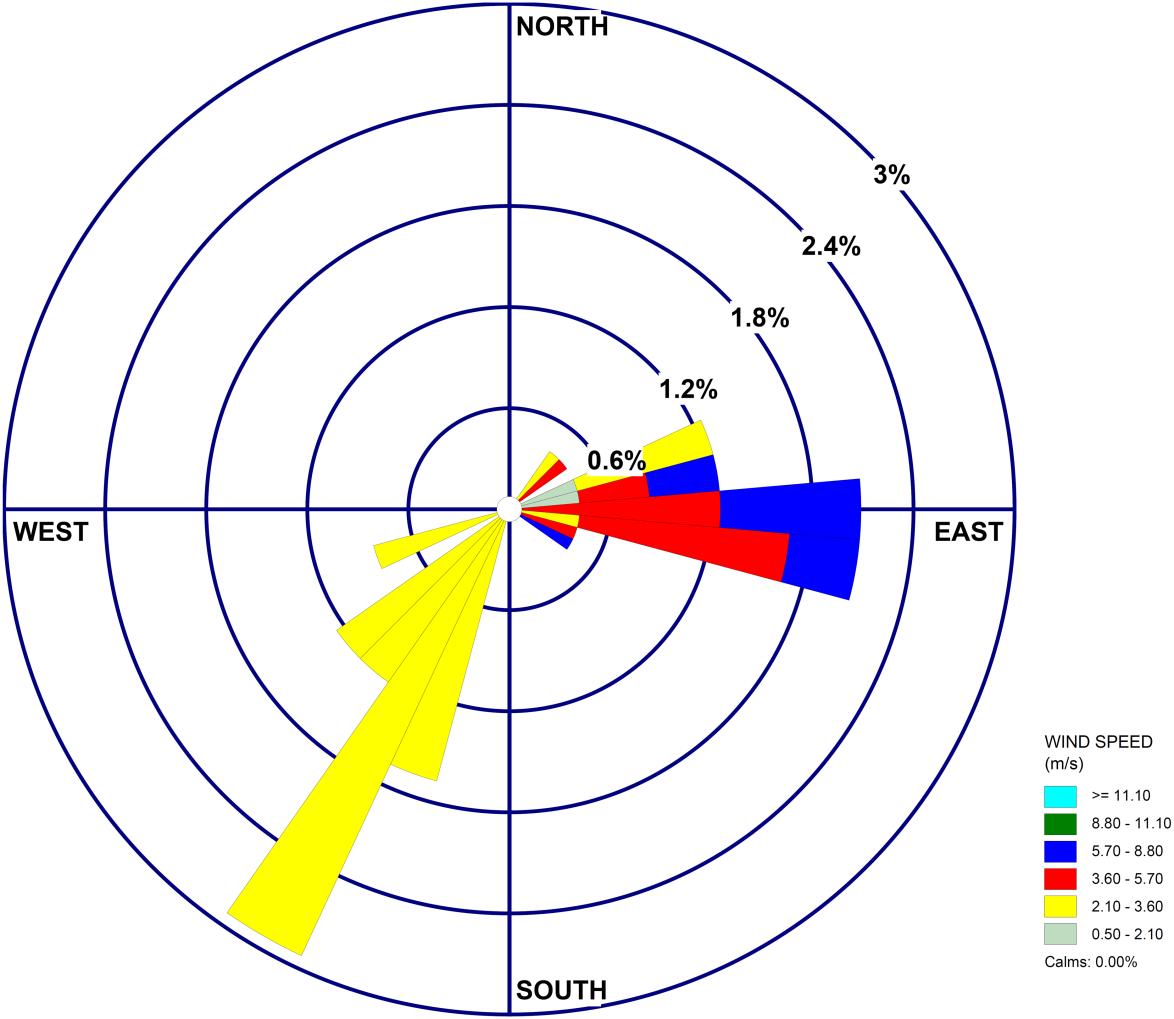
Windrose plot for the period 23 April 2016 to 02 May 2016. Wind speed >=11.10 m/s (Cyan colour), 8.80–11.10 m/s (Green colour), 5.70–8.80 (Blue colour), 3.60–5.70 m/s (Red colour), 2.10–3.60 m/s (Yellow colour), 0.50–2.10 m/s (Light green colour).

Figure 5 shows concentration contours of simulated CO from Uttarakhand at different time steps (24∕04∕2016 2100UTC and 28∕04∕2016 1500UTC) observed at 950 hPa (∼500 m). 24 April was a day with fewer fire counts and hence lesser concentration, whereas on 28 April, the concentrations are high as seen from the contours over Uttarakhand. Figure 5 also shows that the transport of CO during these days is mainly towards north-east from the fire locations as is shown in the wind-rose plot (Fig. 6). The direction marked for the wind is the direction wind is blowing from. The mean wind speed during the period is 3.75 m/s with maximum occurrence towards the north-east direction. Low frequency higher speed easterly wind is diverted to north-north eastern direction by the higher frequency wind from southwesterly wind as seen from the figure.

**Figure 7:**
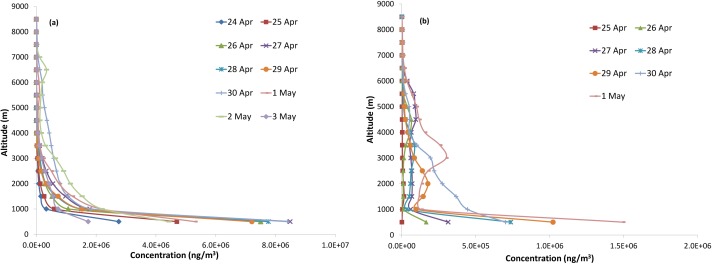
Vertical profile of CO over (A) Uttarakhand during 24 April–03 May, 2016 (B) Tibetan Plateau during 25 April–01 May, 2016. CO concentration profile over Uttarakhand for 24-Apr-2016 (dark blue line with diamond markers), 25-Apr-2016 (red line with square markers), 26-Apr-2016 (dark green line with triangle markers), 27-Apr-2016 (dark purple line with cross markers), 28-Apr-2016 (teal line with star markers, 29-Apr-2016 (orange line with dot markers), 30-Apr-2016 (light blue line with plus markers), 01-May-2016 (link line with half horizontal dash markers), 02-May-2016 (light green line with horizontal dash markers), 03-May-2016 (light purple line with diamond markers).

The vertical profiles of CO were generated using FLEXPART, at the source location and also over different regions along the direction of transport at every 3 h interval. Figure 7 shows average vertical profile of CO over Uttarakhand during 24 April–3 May, 2016 at 0900 UTC and to a distant region of transport. Profiles at 0900 UTC are considered appropriate to compare with Aqua AIRS overpass time at around 1330 local time over Indian sub-continent (0800 UTC). Vertical distribution of CO concentration over Uttarakhand (Fig. 7A) shows a steep decrease (about 10 times) up to 1km altitude than at altitudes from 1 km to 8.5 km above ground. A close examination of the concentration contours at different heights shows significant transport of CO over Quingzang Gaoyuan (Plateau of Tibet), located at an average altitude of 5,000 m above the mean-sea level, which is shown in Fig. 7B. It is seen that an increase in CO concentration is observed at altitudes around 3,000–3,500 m above ground on 30 April and 1 May and at around 2,000–2,500 m on 29 April over this region. This increase in CO over Tibetan plateau is attributed to long range transport of CO from Uttarakhand forest fire areas. In order to confirm the role of Uttarakhand forest fire events in enhancement of CO observed over Tibetan Plateau, further analysis is carried out using NOAA-HYSPLIT back trajectories.

**Figure 8:**
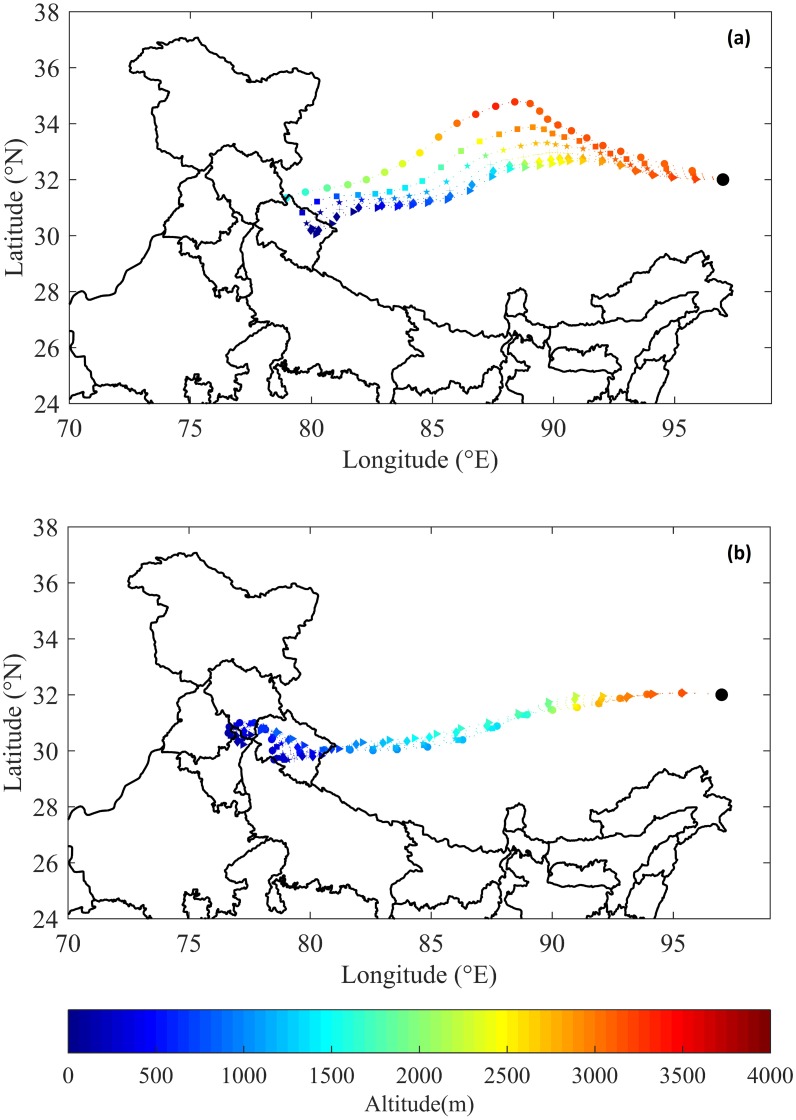
Backward trajectory plots on (A) 01-May 2016 (B) 30-Apr 2016 over Tibetan Plateau (Source: NOAA-HYSPLIT data). (A) 0600 UTC on 01/05/2016 (circle markers), 0500 UTC on 01/05/2016 (square markers), 0400 UTC on 01/05/2016 (star markers), 0300 UTC on 01/05/2016 (plus markers), 0200 UTC on 01/05/2016 (diamond markers), 0100 UTC on 01/05/2016 (triangle markers). (B) 0600 UTC on 30/04/2016 (circle markers), 0500 UTC on 30/04/2016 (square markers), 0400 UTC on 30/04/2016 (star markers), 0300 UTC on 30/04/2016 (plus markers), 0200 UTC on 30/04/2016 (diamond markers), 0100 UTC on 30/04/2016 (triangle markers).

Figures 8A and 8B depict the 3-day back trajectories of Hysplit from 3,200 m altitude over Tibetan Plateau on 01 May and 30 April, 2016 respectively. The black dot indicates the back-trajectories and different start times are shown using different symbols for hours from 0600 UTC to 0100 UTC. The colour gradient depicts the altitudes at respective locations. It is seen that the trajectories indicate that almost 3-day travel time is required for the air-parcel to reach Tibet from Uttarakhand. As seen from Fig. 8, NOAA-HYSPLIT (Stein et al., 2015; Rolph, Stein & Stunder, 2017) backward trajectories from the high concentration location in Tibet shows the three-dimensional transport of air-mass from the ground level at Uttarakhand. The back trajectories show transport of air mass from surface level altitude at Uttarakhand to higher altitudes at Tibetan Plateau, where enhancement in CO concentrations are observed. The air parcel from Uttarakhand at altitudes below 500 m on 28th April, 2016 0600UTC is observed to be reaching Tibet at an altitude of 3,200 m on 1 May, 2016 between 0000 UTC and 0600UTC. Similarly, the air mass from southern Uttarakhand on 28 April, 2016 reaches Tibet at an altitude of 3,200 m on 30 April, 2016 between 0000UTC and 0600UTC. On 28 April, 2016, higher number of fire counts along with favorable wind directions, lead to the transport of more CO towards Tibetan plateau. Thus the HYSPLIT back trajectory analysis corroborates the inference of long-range CO transport from FLEXPART simulations.

## Discussion

Forest fires and their emissions have a huge impact on the concentrations of pollutants in the atmosphere. In order to study the influence of Himalayan forest fire emissions on the atmospheric composition, transport of CO from the 2016 forest fire of Uttarakhand has been investigated in the current research.

The year 2016 saw an increase in the number of forest fires in the state of Uttarakhand and this subsequently led to 45% increase in the columnar CO concentration over the region with respect to 2011–2015 for the period 24 April to 02 May. The study has examined the dispersion of CO from Uttarakhand to surrounding area using a Lagrangian particle dispersion model, FLEXPART. Both columnar and profile concentrations of CO have been simulated using the model and have been compared with AIRS onboard Aqua and show good agreement with coefficient of correlation (r) ∼0.91 over Uttarakhand. The transport of CO was towards the North-East of the state and this led to an increase in the concentration of CO over Tibetan Plateau at 3,000–3,500 m above ground level affecting the air-quality at both local and regional scales.

## Conclusions

In the current study, the long-range transport of CO from Uttarakhand forest fires of 2016 for the duration 24 April–2 May has been studied. High temperature along with dry climatic conditions and dry forest cover contributed to its severity in 2016. During the event period, ∼1,600 forest fire hotspots were recorded. Emissions corresponding to each day were calculated based on the burnt area calculation of each day from hotspots and emission factors for CO per unit area burnt. Forest fires lead to increase in concentrations of air pollutants not only over the source regions, but also over distant locations due to long range transport. Simulation of atmospheric transport of the emissions of CO from the burning during the fire was carried out using a Lagrangian particle dispersion model, FLEXPART. Following are the main conclusions of the present study:

• In Uttarakhand, the higher count of forest fires in 2016 led to higher concentrations of air pollutants including CO (45% increase) compared to previous years.
• CO concentration variation in the profile and column has been simulated well by FLEXPART model runs. Comparison with AIRS onboard Aqua shows good agreement.
• The transport simulation shows trans-boundary effects CO emitted by Uttarakhand forest fires. Long range transport to the Tibetan Plateau is observed, leading to enhancement in CO concentration at higher altitudes, as is observed from the simulations carried out over a distance of ∼3,000 km.

##  Supplemental Information

10.7717/peerj.6507/supp-1 Supplemental Information 1 CO temporal variation with active fire counts during the event (24 April–02 May, 2016) and time series analysis of columnar CO from AIRS

10.7717/peerj.6507/supp-2 Supplemental Information 2 CO profile comparison of AIRS Vs. FLEXPART during the period 24 April–02 May, 2016

10.7717/peerj.6507/supp-3 Supplemental Information 3 Correlation plot of Columnar CO of FLEXPART Vs. AIRS during 24 April–02 May

10.7717/peerj.6507/supp-4 Supplemental Information 4 Vertical profile of CO over (a) Uttarakhand during 24 April - 03 May, 2016 (b) Tibetan Plateau during 25 April–01 May, 2016

## Additional Information and Declarations

### Data Availability

The following information was supplied regarding data availability: Figure 10: NOAA-Hysplit backward trajectory plots on (a) 01/05/2016 (b) 30/04/2016 over Tibetan Plateau has been generated online using the NOAA Air Resources Laboratory (ARL) developed Hysplit model mentioned in the Acknowledgements (http://www.ready.noaa.gov). The raw data are available in the Supplemental Files. The model used in this article, FLEXPART is available at https://www.flexpart.eu/wiki/FpDownloads. The raw data used from the satellite are available at https://giovanni.gsfc.nasa.gov/giovanni/. NOAA Air Resources Laboratory (ARL) developed the Hysplit model available at http://www.ready.noaa.gov.
